# Epidemiology and Natural History of IBD in the Paediatric Age

**DOI:** 10.1155/2014/432807

**Published:** 2014-05-28

**Authors:** Graziella Guariso, Marco Gasparetto, Andrew S. Day, Paul Henderson

**Affiliations:** ^1^Unit of Paediatric Gastroenterology, Department of Women and Children's Health, University Hospital of Padova, Via Giustiniani 3, 35128 Padova, Italy; ^2^Department of Paediatrics, Paediatric Gastroenterology, University of Otago, 8011 Christchurch, New Zealand; ^3^Department of Gastroenterology and Nutrition, Royal Hospital for Sick Children, Edinburgh EH9 1LF, UK

The inflammatory bowel diseases (IBD) are chronic inflammatory conditions of the gastrointestinal tract characterised by episodes of relapse and remission. Although the aetiology of IBD is not fully elucidated, it is thought to be driven by a combination of genetic susceptibility, environmental triggers, and alterations in the gut microbiome that stimulate a dysregulated inflammatory response.

The two main forms of IBD, Crohn's disease (CD) and ulcerative colitis (UC), are diagnosed on the basis of clinical suspicion and laboratory, radiological, endoscopic, and histological findings. It is not always possible to classify patients as having one of these two forms using the diagnostic tools available, with the term inflammatory bowel disease-unclassified (IBD-U) currently used to categorize this subgroup presenting with chronic intestinal inflammation with features of both diseases; many children persist with this diagnosis into adult life. In contrast, the term indeterminate colitis is given to the histopathological description of intestinal inflammation involving bowel resection material.

Both CD and UC can present throughout the age range, including infancy, childhood, and adolescence. The number patients diagnosed with IBD, including childhood, has dramatically increased worldwide over the past 20 years. The reasons behind the rising incidence worldwide are not entirely clear. The current mean prevalence of IBD in the general population of Western countries is estimated at 1/1,000 inhabitants. At present, Scandinavia, Canada, and Scotland have the highest incidences of paediatric IBD worldwide; however, the reason for these high rates remains elusive. Although there are only few epidemiological data available regarding developing countries, the incidence and prevalence of IBD seem to be increasing over the past few decades practically in all regions of the world, indicating its emergence as a global disease.

The changing epidemiology of IBD across time and between geographic areas suggests that environmental factors are likely playing a major role in modifying disease expression. The rising incidence of IBD in developing nations may be linked to industrialization and a Western lifestyle, especially diet.

Insight into the worldwide epidemiology of this disease is, thus, important to identify geographic patterns and time trends. Recent findings will also make it possible to estimate the global public health burden so that health care resources can be allocated and research can be targeted in specific geographic regions. Information about environmental factors hypothetically affecting the incidence and prevalence of this disease may, finally, lead to preventative interventions where possible.

Although less prevalent in infants and in very young children, a number of case reports and small population studies have documented disease onset at very early ages. Underlying immune deficiency and monogenic disorders have been identified in a small subgroup of those diagnosed under the age of 2. An intestinal IBD-like pattern has been found to be associated in these cases with systemic immunodeficiency, within a complex scenario which can even lead to severe life-threatening events. Consequently those under the age of 6 should always have thorough investigation of other aetiologies before a diagnosis of IBD is given.

Assessing the incidence of IBD is complicated, as epidemiological investigations have often ignored cases of IBD-U although they are considered as a distinct disease subtype of IBD. IBD can also be difficult to recognize, particularly in children when extraintestinal symptoms and signs (such as iron deficiency, skin lesions, and liver pathology) are predominant and in third world countries where medical services are lacking.

The aim of this special issue is to review the current levels of knowledge with regard to IBD epidemiology worldwide and to examine its natural history in paediatric patients. The main topics of the issue include the most up-to-date definitions and classifications of paediatric IBD, the epidemiology of IBD worldwide, the epidemiology and natural history of paediatric IBD, and the disease burden within the general and paediatric populations.

We hope that readers of this special issue on paediatric IBD will not only find accurate data and updated reviews, but also consider the questions yet to be resolved such as disease distribution worldwide, its classification, and its impact on the healthcare system.

*Graziella Guariso* *Graziella Guariso* *Marco Gasparetto* *Marco Gasparetto* *Andrew S. Day* *Andrew S. Day* *Paul Henderson* *Paul Henderson*

